# Unveiling GaN Prismatic Edge Dislocations at the Atomic Scale via P-N Theory Combined with DFT

**DOI:** 10.3390/ma18235453

**Published:** 2025-12-03

**Authors:** Li Peng, Lili Huang, Shi Chen, Chengjin Huang, Rui Wang, Mu Li

**Affiliations:** 1 Shenzhen Key Laboratory of Ultra-Intense Laser and Advanced Material Technology, Center for Intense Laser Application Technology, College of Engineering Physics, Shenzhen Technology University, Shenzhen 518118, China; 2300413003@stumail.sztu.edu.cn (L.P.); chenshi@sztu.edu.cn (S.C.); huangchengjin@sztu.edu.cn (C.H.); 2 Institute for Structure and Function, Department of Physics, Chongqing University, Chongqing 400044, China; rcwang@cqu.edu.cn

**Keywords:** gallium nitride, prismatic edge dislocation cores, fully discrete dislocation theory, first-principles calculations

## Abstract

Dislocations in third-generation semiconductor gallium nitride (GaN) have always been a subject of intense study. Here, we investigate the core structures and electronic properties of prismatic edge dislocations in wurtzite GaN using a combination of the discrete Peierls theory and first-principles calculations. We identify four primary analytical core configurations, some of which exhibit reconstruction. Stable glide dislocations are found to be dangling-bond-free, whereas shuffle dislocations typically possess dangling bonds yet exhibit limited electronic activity. Different shuffle-type cores show similar electronic properties, consistent with their structural similarities. The intermediate states during glide dislocation motion may significantly influence GaN’s electronic behavior. This work validates the accuracy of our combined theoretical and computational approach for atomic-scale dislocation characterization and establishes a foundation for dislocation engineering in high-performance GaN devices.

## 1. Introduction

Topological dislocation defects in crystals are often polymorphic and complex at the atomic scale [1] and have a great significant impact on the physical properties of materials. In semiconductor materials, the role of dislocations is particularly pronounced [2,3,4]. However, the growth of GaN is often accompanied by threading dislocations (TDs), primarily caused by lattice mismatches and thermal expansion coefficient differences between GaN and the underlying substrate [2]. The impact of dislocations on the electrical properties of GaN-based devices has been a long-standing topic of interest. Particularly for the most advantageous vertical GaN devices, the mechanisms leading to experimental failures can largely be traced back to the epitaxial growth of the material [5,6,7]. To date, despite recent advances in using graphene to reduce interface slip potential energy and decrease edge dislocation density [8], the dislocation density in GaN epitaxial wafers has not been reduced to levels comparable to those in traditional semiconductor materials like silicon. This persistent challenge continues to impede the full realization of GaN’s potential in semiconductor applications, limiting its widespread adoption and technological development [7]. Although there have been a large number of studies on the dislocations of GaN, its core structures and electrical properties remain inconsistent. Elsner et al. concluded that the edge dislocation electrically inactive with a band gap free from deep levels and mostly exists in the form of saturated core structures [9]. Subsequent research simulated the atomic and electronic structures of GaN dislocations using DFT and revealed that the electrical activity of dislocations is highly dependent on their core structure, while edge dislocations primarily introduced shallow gap states, suggesting a relatively lower impact on electrical properties [10]. Nakano et al. found that both edge dislocations and screw dislocations do not cause the leakage currents in n-type GaN-based devices because no defect level appears near the conduction band bottom [11]. However, later studies demonstrated that dislocations in n-GaN are charged and surrounded by space charge regions [12]. Meanwhile, the exact mechanisms by which dislocations contribute to device degradation remain under debate, the fundamental limitation stems from the fact that the types of dislocations and their associated core structures in GaN at atomic levels remain incompletely understood, and accurately characterizing their atomic-scale configurations continues to persistent challenges.

**Figure 1:**
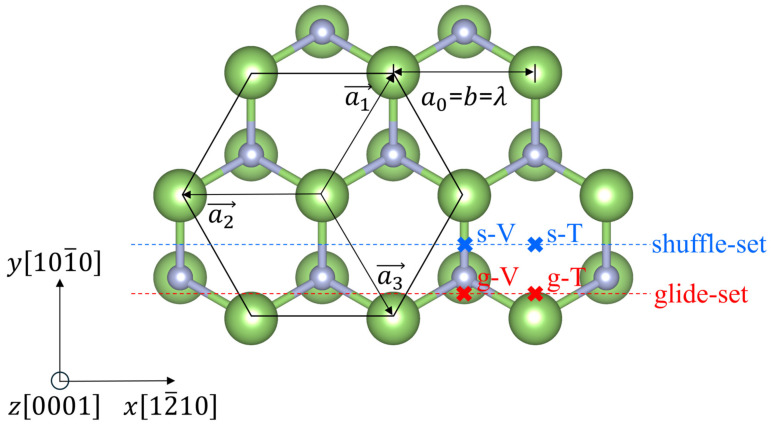
The atomic arrangement of wurtzite GaN perpendicular to the [0001] direction (dislocation line). The slip plane is $\left(10\bar{1}0\right)$, and the direction of the Burgers vector is $\left(1\bar{2}10\right)$. There are two types of layer spacings: the one with a larger layer spacing is called shuffle set and another with a smaller layer spacing is called glide set. g-T and s-T represent the center positions of dislocation at the top of the Peierls energy curves in the glide set and shuffle set, respectively; g-V and s-V represent the dislocation center positions at the valley of the Peierls energy curves in the glide set and shuffle set, respectively.

Generally, GaN exists in the form of a wurtzite structure, including three main types of slip plane, such as basal plane, prismatic plane, and pyramid plane [13]. Depending on the spacing between the slip plane, there are also two slip sets on the prismatic plane, such as the glide set with a smaller interlayer spacing and the shuffle set with a larger interlayer spacing, respectively, as shown in Figure 1. Previous studies of GaN prismatic plane edge dislocations were conducted around three different dislocation core structures [9,11,14,15,16,17,18,19,20,21,22,23,24,25]; they are, respectively, five-to-seven atom ring cores, eight-atom ring cores, and four-atom ring cores, and each core structure has a different dislocation center position. However, previous studies have not been consistent regarding the specific location of their dislocation centers. In 1998, Xin et al. directly observed the eight-atom ring core structure for the first time, and they proposed that edge dislocations do not have deep defect states in the band gap [14]. In 2004, Lymperakis et al. discovered the four-atom ring core structure combining through-focus high-resolution transmission electron microscopy and hierarchical multiscale simulations consisting of the density functional theory, analytical empirical potentials, and continuum elastic theory, and they believed that the four-atom ring core structure does not possess dangling bonds, and the deep defect states are caused by the strain generated by the dislocation and independent of the specific core structure [18], which is contrary to the previously proposed view that the dislocation core structures without dangling bonds are electrically inactive and the electrical activity of dislocations is highly dependent on their core structure. Therefore, it is desirable to systematically study the core structures and related properties of the prismatic dislocations of GaN.

In this work, we applied the fully discrete Peierls theory combined with first-principles calculations based on density functional theory (DFT) to study the edge dislocations in the $\left(10\bar{1}0\right)$ prismatic plane of GaN. Theoretically, we identified four primary analytical core configurations and investigated their core reconstruction structures and also systematically calculated the electrical properties of them to gain deeper insights into the role of edge dislocations in GaN-based devices.

## 2. The Edge Dislocation Core Structures

The renowned Peierls–Nabarro (P-N) model establishes the relationship between dislocation characteristics and mechanical properties in crystals [26,27]. In the classical P-N framework, a perfect crystal is conceptually divided into two semi-infinite parts along the slip plane. The nonlinear interaction between these two parts generates dislocations through topological severe misfit. Originally, this nonlinear interaction was approximated by a sinusoidal function, whose amplitude was determined by imposing an appropriate elastic slope. Subsequently, Lej*č*ek [28] and Kroupa [29] discovered that the γ-surface provides a more accurate description of elastic interactions in body-centered cubic (BCC) structures.

Recently, the fully discrete P-N theory was proposed, which can obtain different balanced dislocation core structures and has been successfully used to investigate the partial dislocations in silicon [30,31], zinc sulfide [32], and the partial dislocations on the basal plane of GaN [33]. The nonlinear interactions are promoted to γ-potential, a function defined on three-dimensional space, and the nonlinear interaction force per unit area is given by a gradient of the γ-potential, f=−∇γ(u). The coordinates used for the equations are shown in Figure 1.

Based on the fully discrete P-N theory, we can obtain the GaN prismatic edge dislocations that satisfy the following equilibrium dislocation equations [30,31,32].

(1) $$\begin{array}{c} -\frac{\beta_{e}}{2\lambda^{2}}\left[\rho_{x}\left(l\right)-\rho_{x}\left(l-1\right)\right]-\frac{K_{ex}}{2\pi \lambda }\sum_{l^{\prime }=-\infty }^{\infty }\frac{\rho_{x}\left(l^{\prime }\right)}{l^{\prime }-l+\frac{1}{2}}=f_{x}\left(l\right),\\ -\frac{K_{e_{y}}}{2\pi \lambda }\sum_{l^{\prime }=-\infty }^{\infty }\frac{\rho_{y}\left(l^{\prime }\right)}{l^{\prime }-l+\frac{1}{2}}=f_{y}\left(l\right), \end{array}$$

mismatch displacement fields, $u_{x}$ is the edge component in the slip plane, and $u_{y}$ is the edge component perpendicular to the slip plane. $f_{x}$ and $f_{y}$ represent the nonlinear interaction forces experienced by the crystal, which can be obtained by the negative gradient of the γ-potential (see the Appendix A) with respect to the displacements $u_{x}$ and $u_{y}$, respectively [30].

According to the dislocation Equation (1), the following energy functional for the dislocation density can be obtained [30,31,32]

(2) $$E=\frac{\beta_{e}}{4\lambda^{2}}\sum_{l=-\infty }^{\infty }\rho_{x}^{2}\left(l\right)-\frac{1}{4\pi \lambda }\sum_{l=-\infty }^{\infty }\sum_{l^{\prime }=-\infty }^{\infty }\left[K_{ex}\rho_{x}\left(l\right)\rho_{x}\left(l^{\prime }\right)+K_{e_{y}}\rho_{y}\left(l\right)\rho_{y}\left(l^{\prime }\right)\right]\psi^{\left(0\right)}\left(\left|l-l^{\prime }\right|+\frac{1}{2}\right)+\sum_{l=-\infty }^{\infty }\gamma \left(u_{x},u_{y}\right),$$

where $\psi^{\left(0\right)}\left(z\right)$ is the Polygamma function [30] and $\lambda =a_{0}=3.247$ Å is the distance between two adjacent paralleled dislocation lines in the slip plane, as shown in Figure 1. $\beta_{e}$ represents the discrete parameter of the lattice [34], while $K_{ex}$ and $K_{ey}$ are energy coefficients of edge components in the slip plane and perpendicular to the slip plane, respectively [13].

According to the fully discrete P-N theory, we obtain that there are two kinds of equilibrium dislocation core structures, respectively, corresponding to the top and valley of Peierls energy curves, for edge dislocations on the glide set and shuffle s-set of the $\left(10\bar{1}0\right)$ slip plane, respectively, and their dislocation center positions are marked in Figure 1. In theory, the energy difference between the two equilibrium dislocation core structures (T-type and V-type) is the Peierls barrier, which is the minimum energy barrier that causes the dislocation to move. With the leading term approximation, the energy per unit length can be expressed as

(3) $$\begin{array}{c} E\left(x_{c}\right)=\frac{1}{2}E_{p}\left(1-\cos\frac{2\pi x_{c}}{\lambda }\right), \end{array}$$

where $E_{p}=\left|E_{T}-E_{V}\right|$, $x_{c}$ represent the center position of the dislocation, and the minimum force causing the dislocation motion can be expressed as

(4) $$\begin{array}{c} \sigma_{p}=\max\left|\frac{1}{b}\frac{dE}{dx_{c}}\right|=\frac{\pi E_{p}}{b\lambda }. \end{array}$$

**Figure 2:**
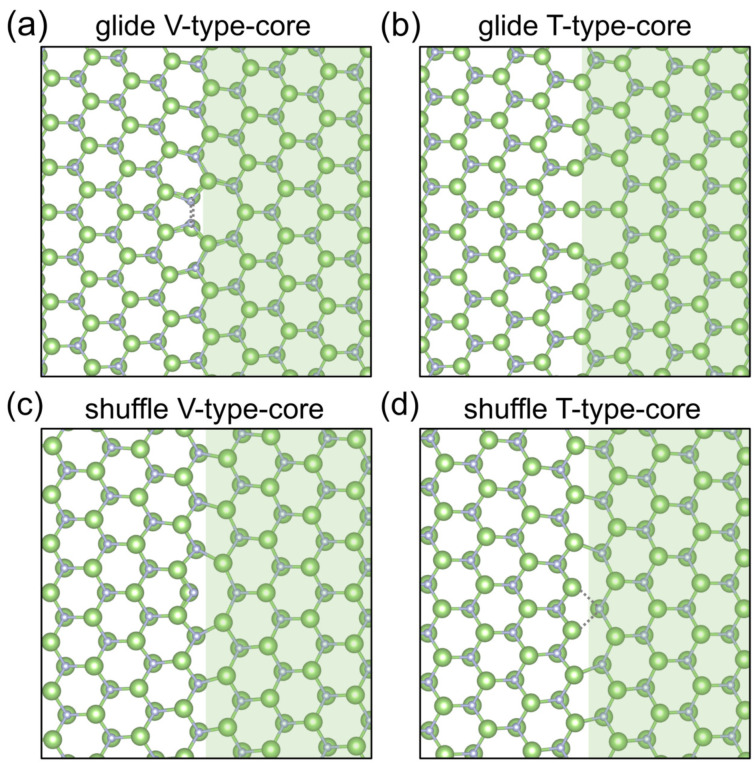
Theoretical dislocation core structures in the glide set and shuffle set of the $\left(10\bar{1}0\right)$ slip plane; here are the projections in the [0001] direction, i.e., the direction of the dislocation line. The boundary of the green shaded area is the slip plane perpendicular to the (0001) plane. (**a**) is the V-type dislocation where the center position at the valley of the Peierls energy curve is in the glide set; (**b**) is the T-type dislocation where the center position at the top of the Peierls energy curve is in the glide set; (**c**) is the V-type dislocation where the center position at the valley of the Peierls energy curve is in the shuffle set; (**d**) is the T-type dislocation where the center position at the top of the Peierls energy curve is in the shuffle set.

**Figure 3:**
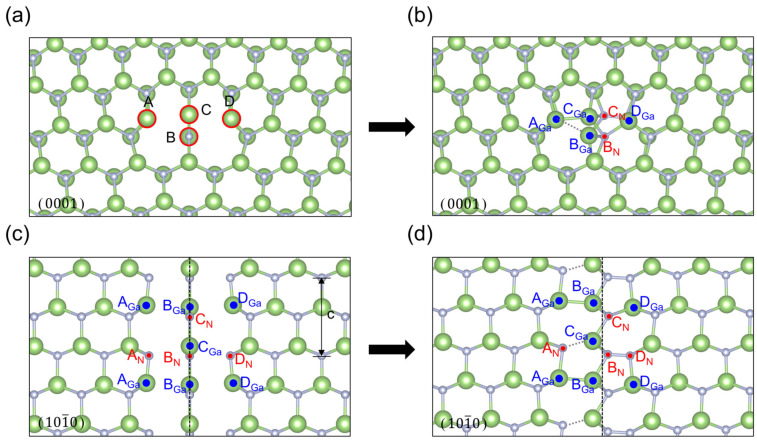
Projections of the theoretical core structure and reconstructed core structure of glide-set T-type dislocation on the (0001) and $\left(10\bar{1}0\right)$ plane: (**a**,**b**) are the projections of the (0001) plane; (**c**,**d**) are the projection of the two layers of atoms above and below the slip plane on the $\left(10\bar{1}0\right)$ plane; (**a**,**c**) are the theoretical core structures; (**b**,**d**) are the reconstructed core structures.

**Figure 4:**
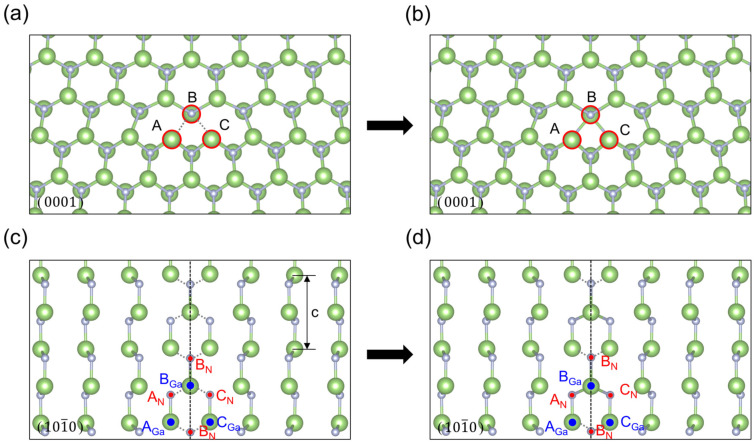
Projections of the theoretical core structure and reconstructed core structure of shuffle-set T-type dislocation on the (0001) and $\left(10\bar{1}0\right)$ plane: (**a**,**b**) are the projections of the (0001) plane; (**c**,**d**) are the projection of the two layers of atoms above and below the slip plane on the $\left(10\bar{1}0\right)$ plane; (**a**,**c**) are the theoretical core structures; (**b**,**d**) are the reconstructed core structures.

**Figure A2:**
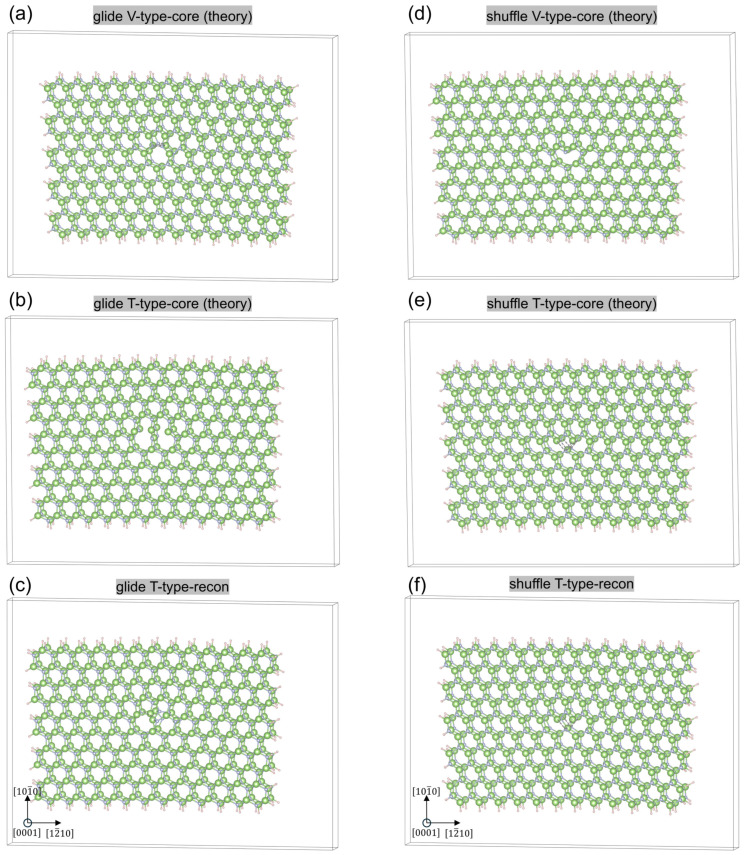
The real structures of the used supercells of the dislocations. The dislocation line is parallel to [0001] and is periodic. The other two directions have a 15 Å vacuum layer, respectively, and the supercell size is 56.83 Å × 42.22 Å × 5.28 Å.

We used first-principles calculations based on DFT to optimize the equilibrium dislocation core structures obtained theoretically, including maintaining the central position of dislocation unchanged and breaking the symmetry of core structures to obtain the reconstruction cores (as shown in Figure 2, Figure 3 and Figure 4). The supercells we used containing 520 and 518 atoms for shuffle and glide dislocations, respectively, and exhibited periodicity along the dislocation line direction. In the other two directions, we had two layers of atoms that are fixed at the boundaries and with a vacuum layer of 15 Å. To address the dangling bonds in the two directions of aperiodic boundary conditions, pseudohydrogen passivation was applied (1.25H for Ga and 0.75H for N). The specific supercells are shown in Figure A2.

The DFT calculations were performed using the Vienna Ab initio Simulation Package (VASP) [35,36]. The interaction between ions and electrons was described using the projected augmented wave (PAW) method [37,38], while the exchange–correlation functional was treated within the generalized gradient approximation (GGA) parameterized by Perdew–Burke–Ernzerhof (PBE) [39]. The wave functions were expanded using plane wave basis sets with a cutoff energy of 450 eV. Brillouin zone sampling was performed using the Monkhorst–Pack scheme, and the k-point was set to 1×1×6. For structural optimization, atomic positions were relaxed using the conjugate gradient algorithm until the force on each atom was less than 0.01 eV/Å. The energy convergence criterion for electronic self-consistent calculations was set to $10^{-5}$ eV.

As shown in Figure 2, we can see that Figure 2a is the dislocation with the center position at the valley of the Peierls energy curve in the glide set, and we call it V-type dislocation, which has a five and seven ring in the core, and there is no dangling bond at the dislocation core, but there is a N-N bond (1.542 Å) and a Ga-Ga bond (2.262 Å) in the per unit period along the dislocation line after the first-principles optimization based on DFT. Figure 2b is the dislocation with the center position at the top of the Peierls energy curve in the glide set, and we call it T-type dislocation, which is a new core structure that should represent an intermediate state of dislocation movement. Meanwhile, the glide-T-type core has more dangling bonds and is in a state of high energy, so a new core reconstruction structure with lower energy can be obtained from this structure, which has a N-N bond (1.523 Å) and a Ga-Ga bond (2.325 Å) in the per unit period along the dislocation line, as shown in Figure 3, and the changes in bond length are shown in the Table 1. Figure 2c is the dislocation with the center position at the valley of the Peierls energy curve in the shuffle set, which has an eight ring in the core. At the center of the dislocation core, there is a nitrogen dangling bond and a gallium dangling bond in the per unit period along the dislocation line direction. Figure 2d is the dislocation with the center position at the valley of the Peierls energy curve in the shuffle set, which looks very similar to the four-ring core structure first discovered by Lymperakis et al. [18]. According to the bond length between gallium and nitride mentioned in ref. [40], this analytical structure seems to have a nitrogen dangling bond and a gallium dangling bond in the per unit period along the dislocation line direction at A, B, and C atomic positions, respectively, as seen in Figure 4, similar to the shuffle-V-type. The bond lengths and bond angles near the dislocation core are shown in Table 1 and Table A5. The geometric positions of all the dislocation cores are shown in Figure 1.

Theoretically, the T-type core is an unstable equilibrium state at the top of energy, and the V-type core is a stable equilibrium state at the valley of energy. Meanwhile, the T-type core of the glide set will spontaneously transform into a V-type core driven by the dangling bonds. Interestingly, the T-type core of the shuffle set directly forms the reconstructed core structure with lower energy driven by the dangling bonds, and the mirror symmetry about the $\left(1\bar{2}10\right)$ plane containing the dislocation line remains unchanged; nevertheless, the atomic configuration along the dislocation line exhibits structural modifications. As shown in Figure 4, along the direction of the dislocation line, we can see that at the B atomic position, there is a gallium atom forming five Ga-N bonds per unit period becoming supersaturated, where the nitrogen atom seems to still have a hanging bond. However, at A and C atomic positions, the nitrogen atoms are four-bond saturated, while the gallium atoms seems to retain their banding bonds, respectively. The changes in bond length are shown in Table 1. We can see that the distance between A_*N*_ and B_*Ga*_ is equal to the distance between C_*N*_ and B*_Ga_*, whether in the theoretical core structure or the reconstructed core structure, the same as for A_*Ga*_-B*_N_* and C_*Ga*_-B*_N_*. However, after the core reconstruction, the length changes from 2.274 Å to 2.195 Å in A_*N*_-B_*Ga*_ and C*_N_*-B_*Ga*_ are similar to those of A*_Ga_*-B_*N*_ and C_*Ga*_-B*_N_*, which went from 2.398 Å to 2.317 Å. Meanwhile, the average bond length of Ga-N ranges from 1.80Å to 2.24 Å [40]. This seems to indicate that B*_Ga_* is more inclined to form a supersaturated five-bond, while B_*N*_ still maintains a dangling bond or has two weaker Ga-N bonds.

## 3. The Electronic Properties of Core Structures

**Figure 5:**
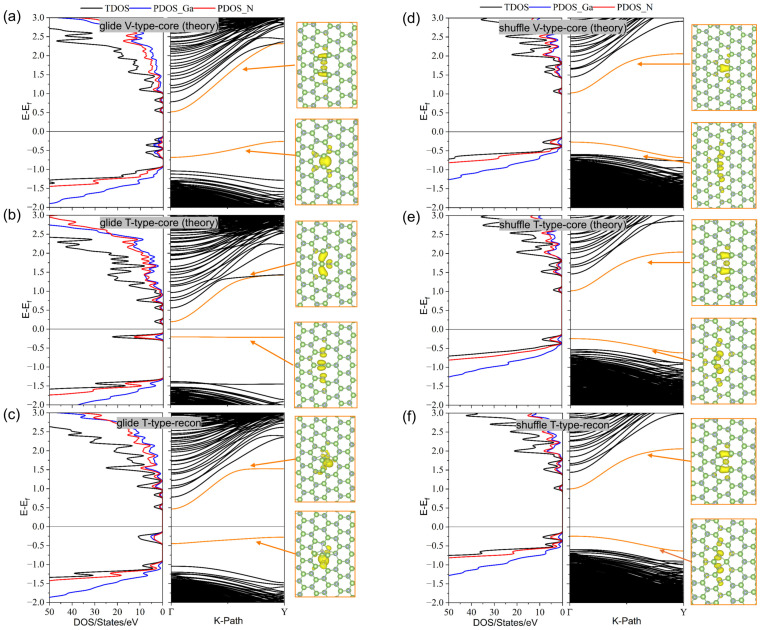
The electronic density of states (DOSs) and energy bands of the dislocation core structures in the $\left(10\bar{1}0\right)$ plane. (**a**–**c**) are the electronic DOSs and energy bands of V-type, T-type, and T-type reconstruction cores of the glide set, respectively; (**d**–**f**) are the electronic DOSs and energy bands of V-type, T-type, and T-type reconstruction cores of the shuffle set, respectively.

**Figure A4:**
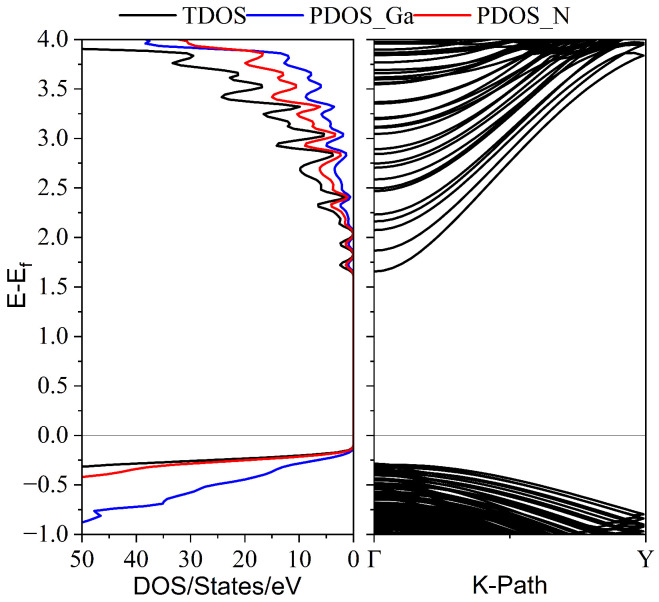
The electronic density of states (DOSs) and energy bands of an ideal supercell.

**Figure A5:**
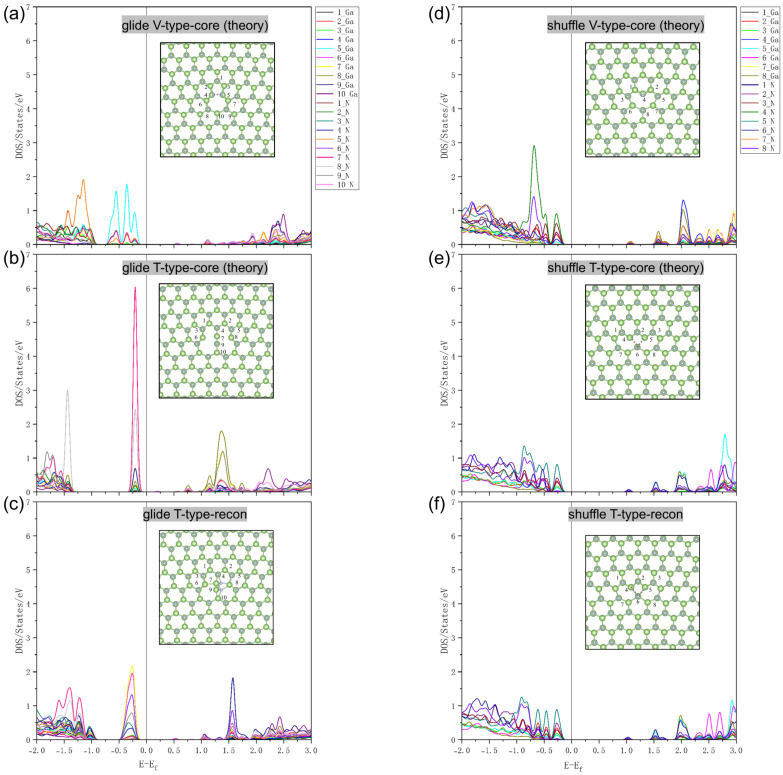
The projected DOSs with labeled atomic contributions. (**a**–**c**) are the projected DOSs of V-type, T-type, and T-type reconstruction cores of the glide set, respectively; (**d**–**f**) are the projected DOSs of V-type, T-type and T-type reconstruction cores of the shuffle set, respectively.

To investigate the impact of the prismatic edge dislocations on the electrical properties of GaN, we analyzed the electronic density of states (DOSs) and energy bands for both glide and shuffle sets compared with the ideal supercell (shown in Figure A4). The electronic DOSs and energy bands of the dislocation core structures of the glide set are shown in Figure 5a–c. We can see in Figure 5a that the V-type core has an occupied state in the band-gap region, which is mainly contributed by the Ga-Ga and N-N bonds at the dislocation center and also includes the contribution of the p orbital of the nitrogen atoms near the dislocation core, and the unoccupied states near the conduction band are mainly provided by the s orbital of nitrogen atoms in the stretched region near the dislocation core. Meanwhile, Figure 5b shows that there is an occupied state below the Fermi level provided by the p orbital of nitrogen atoms in the compression region of the dislocation core and an unoccupied state above the Fermi level provided by the p orbital of nitrogen atoms in the stretched region and gallium dangling bonds in the compression region of the dislocation core, which will enhance the conductivity of GaN. Meanwhile, the unoccupied state originally close to the Fermi level will disappear once the T-type core forms a reconstruction core driven by the dangling bonds, and the unoccupied state near the conduction band after reconstruction is mainly provided by the weaker Ga-N bond, and the occupied states are provided by Ga-Ga bonds and nitrogen atoms around the core, similar to the glide-V-type core structure, as shown in Figure 5c. The electronic DOSs and energy bands of the dislocation core structures of the shuffle set are shown in Figure 5d–f. We can see that the electronic DOSs and energy bands of these three dislocations are similar, with an occupied state near the top of the valence band provided by the p orbital of the nitrogen atoms in the compression region of the dislocation core and an unoccupied state near the conduction band provided by the gallium atoms and the s orbital of nitrogen atoms at the dislocation core. This is because, although the core structures of shuffle-set dislocations are different, the nitrogen and gallium atoms provide interstitial states at their core positions that have similar coordination as well as bond lengths and bond angles, shown in Table 1 and Table A5. Meanwhile, the projected DOSs of the atoms at the dislocation core shown in Figure A5 also validate the above analysis regarding the defect states.

## 4. Discussion and Conclusions

Regarding dislocations in GaN, one of the most widely discussed issues is how these dislocations affect GaN-based devices. Over the years, researchers have held varying perspectives and reached different conclusions. The fundamental reason for this lies in the current inability to accurately describe the core structures of different dislocations in GaN at the atomic scale. Previous studies have often relied on simulation calculations and direct observation using transmission electron microscopy (TEM) to investigate the core structures of dislocations in GaN. However, due to the subjectivity in the choice of computational methods, conditions, and empirical potentials, simulation results can vary significantly. Additionally, the core structures of dislocations in GaN can be altered by the electron beam used in TEM, making it difficult to experimentally observe certain intermediate states that play critical roles in dislocation motion. As a result, it remains challenging to uncover the stability of different dislocation core structures and their universal relationships with the physical properties of materials.

Topological dislocation defects in crystals are often polymorphic and complex at the atomic scale [1]. We applied the fully discrete dislocation theory combined with first-principles calculations based on DFT to study the core structures and electrical properties of prismatic $\left(10\bar{1}0\right)$ plane edge dislocations in wurtzite GaN. Through this theory, we can obtain four different analytical equilibrium core structures for the prismatic edge dislocations in wurtzite GaN: glide-V-type, glide-T-type, shuffle-V-type, and shuffle-T-type. The dislocation centers of T-type and V-type cores differ by half of a λ, which is the distance between adjacent dislocation lines [30], and their specific core positions are shown in Figure 1. In theory, these core structures can undergo core reconstruction under certain perturbations, especially T-type cores. Interestingly, the shuffle-T-type core structure spontaneously forms a reconstructed core, causing the gallium atoms at the dislocation center to adopt a supersaturated five-bond, where the nitrogen atoms still retain the unsaturated three-bond according to the bond length between gallium and nitride mentioned in ref. [40]. This result is different from the previous results which found that there are no dangling bands in the four-ring structure [18]. Despite this, its density of states is similar to that of the shuffle-V-type and the theoretically predicted shuffle-T-type structures. Although the reconstructed core structure of the shuffle-T-type dislocation has passivated some dangling bonds and undergone some symmetry changes, the overall core configuration and bond angles of atoms near the dislocation core remain largely unaltered, and this seems to indicate that, compared with the influence of dangling bonds on the density of states, the bond length and bond angle between atoms seem to play a more decisive role in the density of states; see Table A5. This also precisely shows that our fully discrete Peierls theory comprehensively accounts for lattice discreteness by analyzing the forces on atoms within the glide plane, combined with first-principles calculations based on DFT, enabling the accurate determination of dislocation core structures of the crystal at the atomic level, and also providing a direction for the change in core structure when dislocation moves. Moreover, in the shuffle-set dislocations, these occupied states are predominantly contributed by nitrogen atoms undergoing compressive deformation near the dislocation core, whereas the nitrogen dangling bonds at tensile-strained sites in the shuffle-T-type dislocations provide only minimal unoccupied states, making them easily overlooked. Although it is well-recognized that GGA-PBE pseudopotential tends to underestimate the band gap of wide-gap semiconductors like GaN. However, it is also very meaningful to conduct a qualitative analysis of the electronic properties by comparing them with the results of the ideal supercell (shown in Figure A4). Meanwhile, GGA-PBE provides a computationally efficient and reliable framework for this purpose, especially given the large system sizes involved. The results indicate that both glide-set and shuffle-set dislocations introduce defect states in the band gap. However, the defect states introduced by glide-set dislocations are closer to the Fermi level and are more localized, which may become the recombination centers for electrons and holes, thus having a significant impact on the photoelectric properties of gallium nitride. On the other hand, the occupied and unoccupied states introduced by shuffle-set dislocations are, respectively, close to the valence band and the conduction band and can be regarded as deep energy levels. Their impact on the photoelectric properties of gallium nitride is not so significant. Moreover, the intermediate T-type glide dislocation plays a crucial role in enhancing conductivity through its unoccupied states near the Fermi level. This phenomenon underscores the critical influence of kinetic processes on device performance. While this work has focused on the equilibrium core structures and their electronic properties, the identified polymorphic core configurations provide the atomic-scale basis for understanding dislocation mobility. The experimental observation of room-temperature glide suggests that the energy barriers between these meta-stable cores are likely modest. A quantitative determination of the activation energy for prismatic dislocation slip is a vital and natural extension of this work. Experimental verification through advanced microscopy techniques would provide essential validation of these theoretical predictions.

It is well-known that the movement of dislocations is the microscopic mechanism for the plastic deformation of crystals, and the minimum stress of dislocation movement is Peierls stress. The Peierls stress obtained from the fully discrete Peierls theory is higher than the results in the previous literature, and the theoretical result of our shufflet-set dislocations is basically close to the first-principles calculation result based on DFT. Although it is lower than the result obtained from the nanoindentation experiment, it is still close to it. The simulated energy difference between T-type and V-type dislocations in the glide set is relatively higher. The reason for this is related to the existence of N-N bonds and Ga-Ga bonds in the V-type core structure. However, this energy difference is smaller than the theoretical ultimate shear stress required to generate a dislocation ring through the uniform nucleation mechanism in an ideal crystal, indicating that it is also within a reasonable range. So far, the Peierls theory has not fully considered the nonlinear interaction at the dislocation core, and the interaction among the atoms in each infinite-half part is treated in the harmonic approximation and the nonlinear elasticity is not considered, which might be the reason for the relatively low theoretical results. In future theories, if the strong nonlinear interaction at the dislocation core can be taken into account, core structures that are more in line with the actual situations can be theoretically obtained, including the core reconstruction structures, which can also be directly derived from the theory.

In conclusion, we employed the fully discrete Peierls theory combined with first-principles calculations to investigate the core structures and electrical properties of prismatic edge dislocations in GaN at the atomic level. Our findings reveal that stable glide-set dislocations are dangling-bond-free but contain Ga-Ga and N-N bonds, whereas stable shuffle-set dislocations typically possess dangling bonds. Glide-set dislocations have a greater impact on electrical behavior compared to shuffle-set dislocations. Different shuffle-type cores show similar electronic properties, consistent with their structural similarities. The intermediate states during glide dislocation motion may significantly influence GaN’s electronic behavior. This work validates the accuracy of our combined theoretical and computational approach for atomic-scale dislocation characterization and provided a foundation for optimizing material performance in high-performance GaN devices.

## Figures and Tables

**Table 1:** The bond length changes in the reconstructed core structure; the items marked with * are unbound, and the items marked with ^†^ are suspected of forming bonds.

		A*_Ga_*-A*_N_*	B_*Ga*_-B*_N_*	C*_Ga_*-C*_N_*	A_*N*_-B_*Ga*_	C_*N*_-B_*Ga*_	A_*Ga*_-B_*N*_	C_*Ga*_-B_*N*_	A_*Ga*_-C_*Ga*_	A_*N*_-C_*Ga*_	B_*N*_-D_*N*_	C_*N*_-D_*Ga*_
glide T-type	theory (Å)	1.873	1.867	1.813	-	1.823	-	1.833	2.940 *	3.070 *	2.771 *	3.346 *
	recon (Å)	2.084	2.063	2.025	-	1.946	-	1.940	2.325	2.319 ^†^	1.523	2.063
	Δl (Å)	0.211	0.196	0.212	-	0.123	-	0.107	−0.615	−0.751	−1.248	−1.283
shuffle T-type	theory (Å)	1.933	1.981	1.933	2.274 ^†^	2.274 ^†^	2.398 ^†^	2.398 ^†^	-	-	-	-
	recon (Å)	1.942	2.067	1.942	2.195	2.195	2.317 ^†^	2.317 ^†^	-	-	-	-
	Δl (Å)	0.009	0.095	0.009	−0.079	−0.079	−0.081	−0.081	-	-	-	-

The length of the Ga-Ga bond is 2.74 Å in ref. [18]. The lengths of the Ga-Ga bond and N-N bond are 2.49 Å and 1.57 Å, respectively, in ref. [33]. The lengths of the Ga-Ga bond and N-N bond are 2.18 Å and 1.54 Å, respectively, in ref. [10]. The lengths of the Ga-Ga bond and N-N bond are 2.28 Å and 1.51 Å, respectively, in ref. [41]. The length of the bond is 2.44 Å in bulk Ga [42]. The average length of the Ga-N bond ranges from 1.80 Å to 2.24 Å and the maximum bond length does not exceed 2.40 Å [40].

**Figure A1:**
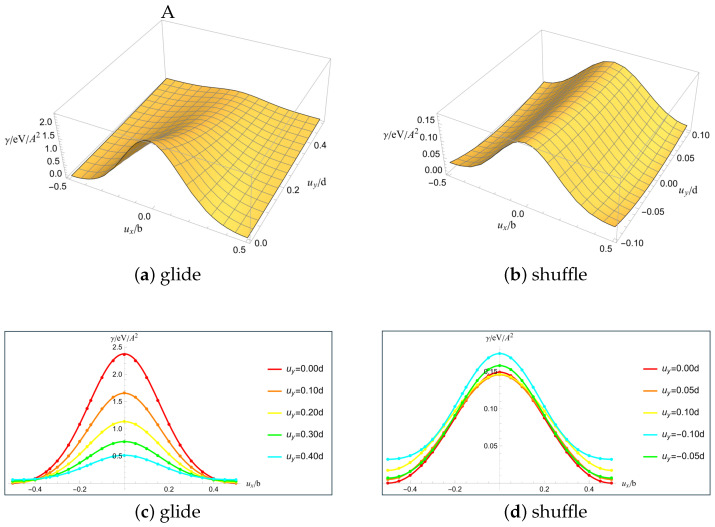
The γ-potential in units of eV/Å^2^ as a function of the relative translation along the $\langle 1\bar{2}10\rangle$ ($u_{x}$) and $\langle 10\bar{1}0\rangle$ ($u_{y}$) direction, $u_{y}$ is the space increase and $d+u_{y}$ is the distance between slip planes, and *d* is the lattice space of the glide set or shuffle set of an ideal crystal. (**a**,**b**) are the energy planes fitted by Equation (A1), (**c**,**d**) are the projection diagrams corresponding to different layer spacings, the solid lines are given by the analytical expression, and the dot lines are given by the first-principles calculations.

**Figure A3:**
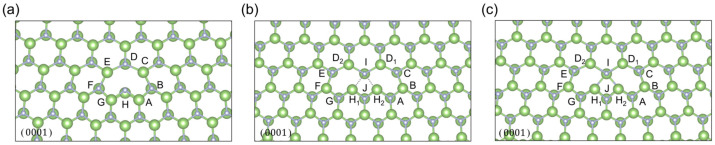
Atomic structure schematics of the (0001) plane showing labeled atoms around three shuffle-set dislocation core structures: (**a**) shuffle V-type (theory), (**b**) shuffle T-type (theory), and (**c**) shuffle T-type-recon.

## Data Availability

The original contributions presented in this study are included in the article. Further inquiries can be directed to the corresponding authors.

## Appendix A. The Fully Discrete Peierls Theory

### Appendix A.1. The γ-Potential of *(*$10\bar{1}0$*)* Slip Plane

The nonlinear interaction between two semi-infinite crystals, formed by cutting a bulk crystal along a slip plane, is quantified by the γ-surface computable via interatomic potentials or DFT. Crucially, atomic relaxation perpendicular to the slip plane is enforced to model dislocation induced interplanar spacing changes. We generalize this framework by introducing a γ-potential that additionally captures energy modulation from out-of-plane displacements, enabling a unified description of slip–plane interactions. The γ-potential is defined as the interaction energy between two parallel half-infinite crystals. When a crystal is cut into two half-infinite parts by a given cut plane, the interaction energy will change when the two parts experience a relatively rigid translation. The interaction energy per unit area as a function of the rigid translation is referred to as the γ-potential of the cut plane. Because a rigid translation can be parallel or perpendicular to the cut plane, it is characterized by a three-dimensional vector. Therefore the γ-potential is a function defined on three-dimensional space. The nonlinear interaction force per unit area, $f=(f_{x},f_{y},f_{z})$, is given by a gradient of the γ-potential.

Figure 1 illustrates the atomic arrangement on the (0001) plane of wurtzite GaN. The vectors ${\vec{a}}_{1}$, ${\vec{a}}_{2}$, and ${\vec{a}}_{3}$ denote the three base vectors of the basal plane in the hexagonal close-packed structure. As shown in Figure 1, there are two types of layer spacings on the $\left(10\bar{1}0\right)$ plane, one with a larger layer spacing is called the shufflet set, and another with a smaller layer spacing is called the glide set. The γ-potential for the edge dislocation on the $\left(10\bar{1}0\right)$ plane is calculated in the following way: Firstly, selecting four points along the direction of the Burgers vector, $u_{x}=0,\;u_{x}=b/4,\;u_{x}=b/3,\;u_{x}=b/2$; secondly, for each individual translation, calculating the energy variation as the space increasing; thirdly, by using the data calculated, and the method is the same as that in ref. [30]. The $\gamma_{i}\;\left(i=0,\;1,\;2,\;3\right)$ as a function of the perpendicular displacement (space change) are determined by fitting. It is found that the γ-potential numerically calculated from the first principles can be well-described by the following analytical expression, and the calculated γ-potential is shown in Figure A1.

(A1) $$\begin{array}{cccc} \; & \; & \gamma_{b}\left(u_{x},u_{y}\right) & =\phi_{0}+\gamma_{1}\left(u_{y}\right)\cos^{2}\frac{\pi u_{x}}{b}+\gamma_{2}\left(u_{y}\right)\cos^{4}\frac{\pi u_{x}}{b}+\gamma_{3}\left(u_{y}\right)\cos^{6}\frac{\pi u_{x}}{b},\\ \; & \; & \gamma_{1}\left(u_{y}\right) & =\frac{1}{3}\left(-13\phi_{0}+36\phi_{1}-32\phi_{2}+9\phi_{3}\right),\\ \; & \; & \gamma_{2}\left(u_{y}\right) & =2(3\phi_{0}-14\phi_{1}+16\phi_{2}-5\phi_{3}),\\ \; & \; & \gamma_{3}\left(u_{y}\right) & =-\frac{8}{3}\left(\phi_{0}-6\phi_{1}+8\phi_{2}-3\phi_{3}\right), \end{array}$$

where $u_{y}$ is the change in displacement perpendicular to the slip plane, $\phi_{i}$ is the Morse function as follows

(A2) $$\begin{array}{c} \phi_{i}\left(u_{y}\right)=s_{i}\left(e^{-2\alpha_{i}\left(u_{y}-\upsilon_{i}\right)/d}-2e^{-\alpha_{i}\left(u_{y}-\upsilon_{i}\right)/d}\right)+t_{i},\;\;i=0,1,2,3. \end{array}$$

with the relevant parameters of this formula are given in Table A1, where *d* is the layer spacing of the slip plane.

**Table A1:** The parameters in the Morse function.

	$s_{0}$	$s_{1}$	$s_{2}$	$s_{3}$	$\alpha_{0}$	$\alpha_{1}$	$\alpha_{2}$	$\alpha_{3}$	$v_{0}$	$v_{1}$	$v_{2}$	$v_{3}$	$t_{0}$	$t_{1}$	$t_{2}$	$t_{3}$
glide	0.980	0.801	0.805	0.841	0.739	0.997	1.098	1.189	0	0.567	0.682	0.757	0.980	0.954	0.978	1.027
shuffle	0.238	0.209	0.175	0.126	3.122	2.623	2.435	2.346	0	−0.017	0.021	0.124	0.238	0.273	0.278	0.271

By fitting the energy variations caused by displacements both on and perpendicular to the slip plane, we obtained the final generalized stacking fault energy, shown in Figure A1. Our findings indicate that the γ-potential, as calculated from first principles, can be effectively described by analytical Equation (A1), with all relevant parameter values listed in Table A1. As shown in Figure A1, the relationship between the γ-potential of the shuffle set and the interlayer spacing indicates that the energy minima during lattice sliding occur near the ideal interlayer spacing. Therefore, changes in dislocation configurations concerning the shuffle set caused by variations in interlayer spacing can be considered negligible, but the variation in the spacing of the glide set cannot be ignored.

### Appendix A.2. The Relevant Parameters of the Dislocation Equation

According to the isotropic approximation, the discrete parameter $\beta_{e}$ and the energy factors $K_{e_{x}}$ and $K_{e_{y}}$ mentioned in Equation (1) are given by the following expressions [13,34]:

(A3) $$\begin{array}{cccc} \; & \; & K_{ex} & =\left[{\left(C_{11}C_{33}\right)}^{1/2}+C_{13}\right]{\left\{\frac{C_{44}\left[{\left(C_{11}C_{33}\right)}^{1/2}-C_{13}\right]}{C_{33}\left[{\left(C_{11}C_{33}\right)}^{1/2}+C_{13}+2C_{44}\right]}\right\}}^{1/2},\\ \; & \; & K_{ey} & =\left[{\left(C_{11}C_{33}\right)}^{1/2}+C_{13}\right]{\left\{\frac{C_{44}\left[{\left(C_{11}C_{33}\right)}^{1/2}-C_{13}\right]}{C_{11}\left[{\left(C_{11}C_{33}\right)}^{1/2}+C_{13}+2C_{44}\right]}\right\}}^{1/2},\\ \; & \; & \beta_{e-glide} & =\frac{\left(C_{11}-C_{12}\right)a_{0}^{3}}{16\sigma },\\ \; & \; & \beta_{e-shuffle} & =\frac{\left(3C_{11}+5C_{12}\right)a_{0}^{3}}{24\sigma } \end{array}$$

where σ is the primitive cell area of the $\left(10\bar{1}0\right)$ slip plane and $C_{11},C_{12},C_{13},C_{33}$, and $C_{44}$ are elastic constants, and the values come from our previous work [33].

To characterize the dislocation, we employed an energy minimization approach. This method involves solving the dislocation equation to determine the configuration that yields the minimum energy value, thereby revealing the characteristic parameters of the system. The specific values of these parameters are presented in Table A2, providing a quantitative foundation for our subsequent analysis. With these characteristic parameters established, we were able to proceed to determining the mismatch field induced by the dislocation.

The solutions of the discrete dislocation equations (Equations (1) and (2)) are proposed to be [30]

(A4) $$\begin{array}{c} u_{x}\left(l\right)=\frac{\hat{D}u_{x}^{\left(0\right)}}{\hat{D}Q_{0}}b,\;\;\;u_{y}=\frac{c_{y}b\xi }{\xi^{2}+{\left(l-\alpha_{y}\right)}^{2}\lambda^{2}}, \end{array}$$

where $\hat{D}$ is the parametric derivative

$$\hat{D}=\left(1-c_{x}\right){\bar{\zeta }}^{2}-c_{x}{\bar{\zeta }}^{3}\frac{\partial }{\partial \bar{\zeta }},$$

and

$$u_{x}^{\left(0\right)}\left(l\right)=\frac{i}{2\bar{\zeta }}\left[\psi^{\left(0\right)}\left(-l+\alpha_{x}-i\bar{\zeta }+1\right)-\psi^{\left(0\right)}\left(-l+\alpha_{x}+i\bar{\zeta }+1\right)\right],$$

$$Q_{0}=\frac{\pi }{\bar{\zeta }}\times \frac{\sinh(2\pi \bar{\zeta })}{\cosh\left(2\pi \bar{\zeta }\right)-\cos\left(2\pi t\right)}\;\;\;\;\bar{\zeta }=\frac{\zeta }{\lambda }.$$

In the expression of the solution, $\alpha_{x}=0,1/2$ and $\alpha_{y}=1/2-\alpha_{x}$, labeling the center position of the dislocation distribution, and can be labeled by V-type ($\alpha_{x}=0$) and T-type ($\alpha_{x}=1/2$). ζ and ξ describe characterized width. $c_{x}$ and $c_{y}$ are structure constants. Using the expression Equation (A4) as the trial function with the variational parameters ζ, ξ, $c_{x}$, and $c_{y}$, the approximated solutions are obtained from the Ritz variational method. The final results are shown in Table A2, where all the parameters in the solutions of the glide-set and shuffle-set dislocations are listed. The dislocation current density $\rho_{x}$ in Equation (A4) is continuous in the limit λ→0. It is observed that for the narrow dislocation, the characteristic length ζ should be renormalized by a factor. For the V-type dislocation, the characteristic length ζ should be replaced by $\zeta^{\prime }=0.68\zeta$ and $\zeta^{\prime }=0.883\zeta$ for glide and shuffle dislocations, respectively, in the continuous approximation. The characteristic length ζ of the T-type dislocation is relatively large and the renormalized factor equals one, $\zeta^{\prime }=\zeta$. Referring to the displacement fields in refs. [30,31], we can obtain the core structures of the glide-set and shuffle-set dislocations, respectively.

**Table A2:** The parameters in the dislocation solution and the energy *E*. ΔE represents the energy difference between the T-type and V-type in theory.

		$\alpha_{x}$	$\alpha_{y}$	$c_{x}$	$c_{y}$	ζ	ξ	$\zeta^{\prime }$	*E* (eV/Å)	ΔE (eV/Å)
glide	V-type	0	1/2	0.783	0.137	0.518	0.294	0.352	1.475	0.016
	T-type	1/2	1	0.886	0.220	0.573	0.360	0.573	1.491	
shuffle	V-type	0	1/2	0	-	0.712	-	0.629	0.753	0.058
	T-type	1/2	1	0.360	-	1.113	-	1.113	0.811	

### Appendix A.3. Energy of the Dislocation Core Structures

The supercells of different dislocation core structures used in the simulation calculation are shown in Figure A2, and Table A3 displays their energies. It includes the energies of theoretically calculated core structures and reconstructed structures for both V-type and T-type configurations under two different slip modes: glide set and shuffle set. The ΔE in the table represents the energy difference obtained by subtracting the energy of the lower-energy V-type from the higher-energy T-type, with units of eV/Å. It can be observed that the shuffle V-type structure has the lowest energy, which is different from the previous research results which held that the energy of the shuffle T-recon structure was the lowest [18]; this should be related to the fact that our results show that this structure has dangling bonds. Theoretically, the energy difference between T-type and V-type is the Peierls barrier, as shown in Table A2. Since the fully discrete dislocation equation still considers linear interactions, while the interaction at the dislocation core is nonlinear, the dislocation core structures obtained from the dislocation core parameters can relatively accurately obtain the atomic distribution in the near field and far field and provide the definite dislocation core positions and the initial force balance model of the dislocation cores. This might also be the reason why our Peierls stress is higher than the experimental value. After optimization through the first-principles calculations based on DFT, generally, the energy of the core structure is reduced. However, after the V-type of the glide set is optimized, N-N bonds and Ga-Ga bonds are formed, thus obtaining a much lower energy than the theoretical analysis result. Therefore, at this time, there would be a significant difference between the DFT energy difference of the V-type and T-type structures and the theoretical energy difference. It can be seen from Table A2, Table A3 and Table A4 that the DFT results of shuffle-set dislocations are similar to the theoretical results because on the premise of ensuring the force-balanced analysis structure, neither V-type nor T-type forms chemical bonds to significantly reduce the energy of the theoretical core structure. The Peierls stresses of the prismatic edge dislocations are shown in Table A4. The yield stress described in ref. [43] is 11.5 GPa. According to Schmidt’s law and with the Schmidt factor in ref. [43] being 0.433, the critical stress for dislocation motion can be obtained as 4.98 GPa, which is higher than the theoretical result. Ref. [44], through the theoretical calculation of the uniform nucleation mechanism of ideal crystals, obtained that the upper limit value of stress for the plastic deformation of GaN is 24.93 GPa.

**Table A3:** The dislocation core energy, and the unit is eV/Å.

		$E_{V}$ (eV/Å)	$E_{T}$ (eV/Å)	ΔE (eV/Å)
glide	theory	-	−650.736	0.379
	recore	−651.115	−650.880	0.234
shuffle	theory	−653.247	−653.178	0.069
	recore	-	−653.243	0.005

**Table A4:** The Peierls stress ($\sigma_{p}$), except for the data marked with *, and the unit is eV/Å^3^ (GPa).

	This Work		Ref. [3]	Ref. [43]	Ref. [44]
theory	glide-set	0.005 (0.741)	0.0013 (0.2)	0.001 (0.15)	0.001 (0.1528)
	shuffle-set	0.017 (2.790)			
DFT	glide-set	0.113 (18.066)	-	-	-
		0.070 (11.175)			
	shuffle-set	0.021 (3.301)	-	-	-
		0.001 (0.217)			
experiment	-		0.0014 (0.23)	0.072 (11.5) *	-

* These data are the yield stresses obtained from the nanoindentation experiment.

### Appendix A.4. The Bond Angles of the Dislocation Core Structure of the Shuffle Set

To elucidate the electronic structure of the shuffle set, we analyzed the atomic bond angles near three distinct dislocation core types. The atoms surrounding each dislocation core were systematically labeled in Figure A5, and their bond angles were measured. Table A5 presents these measurements, with each entry representing the angle formed between an atom and its two adjacent neighbors.

**Table A5:** The bond angles at the positions of different atomic columns.

		A	B	C	D_1_	D_2_	E	F	G	H_1_	H_2_	I	J
Ga	shuffle V-theory (°)	95.164	99.480	129.417	D = 120.743		130.245	99.113	94.772	H = 105.145		-	-
	shuffle T-theory (°)	98.570	99.064	124.868	114.633	114.633	124.868	99.064	98.570	98.649	98.649	113.031	93.847
	shuffle T-recon (°)	100.902	100.166	122.813	114.883	114.833	122.813	100.166	100.902	98.229	98.229	109.897	88.841
N	shuffle V-theory (°)	95.709	96.861	126.929	D = 122.440		127.627	96.306	95.258	H = 100.936		-	-
	shuffle T-theory (°)	98.224	97.965	123.815	116.738	116.738	123.815	97.965	98.224	97.427	97.427	110.859	95.715
	shuffle T-recon (°)	100.885	99.030	121.785	116.876	116.876	121.785	99.030	100.885	97.557	97.557	109.550	88.066
